# Sensitizing leukemia stem cells to NF-κB inhibitor treatment *in vivo* by inactivation of both TNF and IL-1 signaling

**DOI:** 10.18632/oncotarget.14220

**Published:** 2016-12-26

**Authors:** Jing Li, Andrew Volk, Jun Zhang, Joseph Cannova, Shaojun Dai, Caiqin Hao, Chenglong Hu, Jiewen Sun, Yan Xu, Wei Wei, Peter Breslin, Sucha Nand, Jianjun Chen, Ameet Kini, Jiang Zhu, Jiwang Zhang

**Affiliations:** ^1^ Department of Biology, College of Life and Environment Science, Shanghai Normal University, Shanghai, 200234, People's Republic of China; ^2^ Oncology Institute, Cardinal Bernardin Cancer Center, Loyola University Chicago, Maywood, IL 60153, USA; ^3^ Department of Biology, Loyola University Chicago, Chicago, IL 60660, USA; ^4^ Department of Molecular and Cellular Physiology, Loyola University Medical Center, Maywood, IL 60153, USA; ^5^ Department of Cancer Biology, University of Cincinnati College of Medicine, Cincinnati, OH 45219, USA; ^6^ Department of Pathology, Loyola University Medical Center, Maywood, IL. 60153, USA; ^7^ State Key Laboratory for Medical Genomics and Shanghai Institute of Hematology and Collaborative Innovation Center of Hematology, Rui-Jin Hospital, Shanghai Jiao-Tong University School of Medicine, Shanghai, People's Republic of China

**Keywords:** leukemia stem cell, NF-kappa B, IL-1, TNF, JNK

## Abstract

We previously reported that autocrine TNF-α (TNF) is responsible for JNK pathway activation in a subset of acute myeloid leukemia (AML) patient samples, providing a survival/proliferation signaling parallel to NF-κB in AML stem cells (LSCs). In this study, we report that most TNF-expressing AML cells (LCs) also express another pro-inflammatory cytokine, IL1β, which acts in a parallel manner. TNF was produced primarily by LSCs and leukemic progenitors (LPs), whereas IL1β was mainly produced by partially differentiated leukemic blasts (LBs). IL1β also stimulates an NF-κB-independent pro-survival and proliferation signal through activation of the JNK pathway. We determined that co-inhibition of signaling stimulated by both TNF and IL1β synergizes with NF-κB inhibition in eliminating LSCs both *ex vivo* and *in vivo*. Our studies show that such treatments are most effective in M4/5 subtypes of AML.

## INTRODUCTION

Acute myeloid leukemia (AML) is a common hematopoietic malignancy in adults. Incidence is increased in aged populations with average age of 67 years at diagnosis. Intensive chemotherapy is the standard treatment for AML (except for M3 subtype), which can induce complete remission in 40-70% of patients. However, such treatment cannot eliminate AML stem cells (LSCs) in almost all cases, and the remaining LSCs, described as minimal residual diseases (MRD), can reconstitute the tumor by producing new leukemic progenitors (LPs) and leukemic blasts (LBs). Thus, almost all AML patients will succumb to disease relapse due to drug-resistant LSCs and their progeny. Currently, 5-year overall survival for AML patients is 40 to 45% for patients under 60 years of age, and <10% for patients 60 years of age and older [1–4]. In addition, intensive chemotherapy is limited in older patients due to life-threatening inflammation-mediated complications and side effects [5, 6]. Therefore, the development of more specific and efficient targeting strategies for eliminating LSCs in AML treatment is urgent.

NF-κB is a key transcriptional regulator of inflammatory cytokine-stimulated signaling and plays a central role in the development and progression of inflammation-associated cancer [7–10]. NF-κB is also required for maintenance of LSCs. In AML, tumor necrosis factor-α (TNF hereafter) and NF-κB promote LSC survival and proliferation in a feed-forward manner [11]. While NF-κB activity is undetectable in unstimulated normal CD34^+^ hematopoietic stem/progenitor cells (HSPCs), it is constitutively activated in CD34^+^CD38^-^ LSCs isolated from almost all AML patients [12–14]. Also, inactivation of NF-κB signaling selectively eradicates LSCs *in vitro* especially when combined with chemotherapeutic drugs without significant influence on the survival and growth of normal HSPCs [15]. This suggests that targeting NF-κB could be an effective treatment paradigm for eliminating LSCs. However, the anti-leukemia effects of NF-κB inhibition in clinical patients are inadequate, suggesting a protective mechanism exists within the bone marrow environment.

We reported that in many subtypes of AML, especially in M4 and M5, the anti-leukemia effects of NF-κB inhibition are attenuated by autocrine TNF stimulation of JNK (a survival/proliferation signal in LCs) and paracrine TNF stimulates a JNK-mediated necroptotic/apoptotic signal in HSPCs [16]. We determined that inhibition of TNF-JNK signaling provided improved treatment for TNF-expressing AML when combined with NF-κB inhibitors. We also found that co-inhibition of JNK and NF-κB signaling was also effective in some TNF non-expressing LCs and patient samples, suggesting that other cytokines might be secreted by LCs which can also activate JNK signaling in addition to TNF [16].

In this study, we found that in addition to TNF, most LCs, especially M4 and M5 LCs, also secrete interleukin 1β (IL1β). IL1β stimulation of both NF-κB and JNK signaling protects LSCs and LPs from NF-κB inhibition by compensating TNF signaling. Our study suggests that inhibition of both TNF and IL1β signaling could represent an improved treatment for inflammatory cytokine-secreting AML when combined with an NF-κB inhibitor.

## RESULTS

### TNF signaling inactivation only slightly potentiated the anti-leukemic effects of NF-κB inhibitor *in vivo*

**Figure 1:**
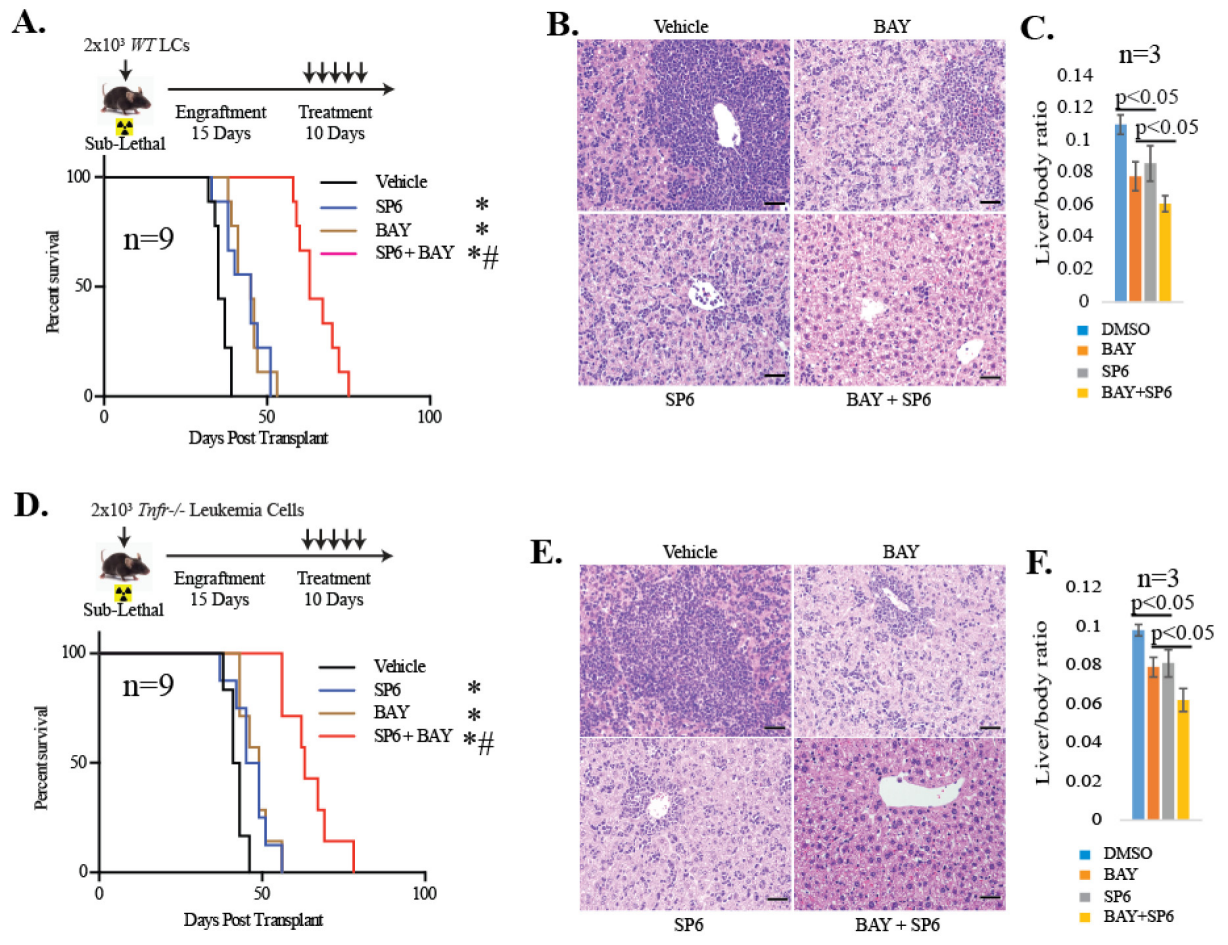
Inactivation of TNF failed to sensitize LCs to NF-κB inhibition *in vivo* WT LCs **A-C.** and *Tnfr*^−/−^ LCs **D-F.** were transplanted into sub-lethally irradiated mice. Schematic diagram of the experimental procedures **(A, D).** On day 15 post-transplantation, mice were randomly divided into four groups and were treated with vehicle, 30 mg/kg SP6 or 10mg/kg BAY individually or in combination daily for 10 days. Livers were collected from recipient mice on day 30 post-transplantation for *WT* LCs (**B-C**) and on day 40 post-transplantation for *Tnfr*^−/−^ LCs (E-F). Three mice from each group were analyzed. Infiltration of LCs was examined by H & E staining of liver sections. The ratio of liver/body weight was presented. Bars, 200μM. Survival of the recipient mice was analyzed by Kaplan-Meier survival graphing (**A, D**). Numbers of mice used in each group were noted. Leukemia was confirmed at the time of death of each transplanted mouse. * indicates p<0.05 when compared to vehicle-treated control. # indicates p<0.05 when compared to Bay or SP individual inhibitor-treated groups.

We reported that TNF stimulates JNK signaling to protect LCs from NF-κB inhibition as shown in primary patient samples, human AML cell lines and *MLL-AF9* (*MA9*)-transduced murine LCs. Inhibition of either TNF or JNK could significantly increase the sensitivity of LCs to NF-κB inhibitor treatment *in vitro* [16]. Consistent with this observation, we found that combined treatment with both the NF-κB inhibitor BAY11-7802 (BAY hereafter) and the JNK inhibitor SP600125 (SP hereafter) profoundly reduced the tumor burden and prolonged the survival of leukemic mice developed from *MA9* transduction (Figure 1A-1C). Currently, there are no clinically available JNK inhibitors approved for use in human subjects; however many TNF blockers have been developed for the clinical treatment of inflammatory diseases such as arthritis [17]. Therefore, we tested whether inhibition of TNF can also sensitize LCs to NF-κB inhibition *in vivo* by transplanting *Tnfr^−/−^* LCs (genomic deletion of *Tnfr1/2*) into sub-lethally irradiated mice and treating with NF-κB inhibitor – (Figure 1D-1F). Consistent with the results of our previous study [16], mice which had received *Tnfr^−/−^* LCs required longer latency for leukemia development than mice which had received *WT* LCs (Supplementary Figure 1). We found that, compared to the vehicle-treated group, NF-κB inhibition was able to slightly extend the lifespan of mice which had received *Tnfr^−/−^* LCs, while a combination of JNK or NF-κB inhibitors also significantly reduced the tumor burden and prolonged the life of *Tnfr^−/−^* leukemic mice (Figure 1D–1F). These data suggested that in addition to TNF, other cytokine(s) might also protect LCs from NF-κB inhibition *in vivo* by stimulating JNK.

### The sensitivity of LSCs and LPs to NF-κB inhibition in culture was dependent on cell density

Using purified LSCs, which are normally cultured in *in vitro* studies at a relatively low density, previous studies demonstrated that LSCs are highly sensitive to NF-κB inhibitor treatment [12, 13]. In our previous studies, we also cultured LCs at a relatively low density (1-2×10^5^/ml). To examine the responses of LSCs and LPs to NF-κB inhibition, we used an unpurified mixed population of LCs containing LSCs, LPs and partially differentiated LBs in our studies because we believed that such a mixture of cells would be more representative of the real situation of LCs in patient bone marrow tissues. *MA9*-transduced murine LCs were used as a model system in our studies because LPs and LSCs among such LCs can be reliably evaluated by colony-forming unit (CFU) assay and *in vivo* transplantation assay, respectively [16, 18].

**Figure 2:**
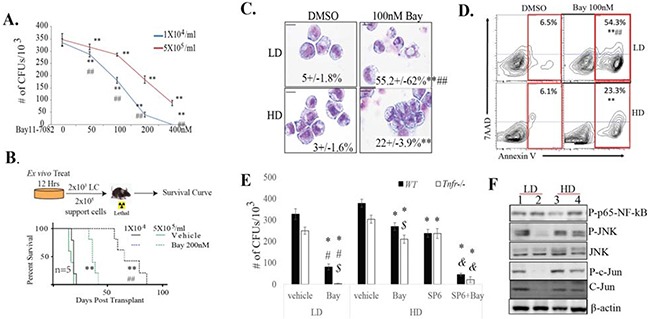
Inactivation of TNF failed to sensitize LSCs and LPs to NF-κB inhibition *in vitro* when cultured at high density *WT LCs* were cultured at low density (1×104/ml, LD) or high density (5×105/ml, HD) and treated with indicated concentrations of BAY for 12 hours. Cells were collected for colony-forming assay, **A.** transplanted into recipient mice for leukemia development **B.,** cell morphology (the numbers presented in each photo are the percentage of morphologically apoptotic cells) C., or cell death by Annexin-V/7AAD staining **D.** The numbers of cells seeded per plate or transplanted per mouse were based on the number of initial treated cells. Results shown are indicative of three independent trials in **(A).** Five mice were used in each treatment group in (B). ** indicates p<0.01 when compared to vehicle controls. ## indicates p<0.01 when compared to HD groups with the same dosage of BAY. **E.** *WT* and **Tnfr*^−/−^* LCs were incubated at LD (1×104/ml) or HD (5×105/ml) and were treated with 200nM Bay, 10μM SP6 or both in combination for 12 hours and then collected for CFU assay. Vehicle-treated cells were used as controls. * indicates p<0.05 when compared to vehicle-treated controls. $ indicates p<0.05 when compared to *WT LCs*. # indicates p<0.05 when compared to HD groups with same treatments. & indicates p<0.05 when compared to HD groups with Bay or SP individual treatment. **F.** *WT* (lanes 1 and 3) and *Tnfr*−/− LCs (lanes 2 and 4) were incubated at LD (1×104/ml) or HD (5×105/ml) for 24 hours. JNK and NF-κB signaling activity were compared by Western blotting.

To test whether cell density influences the response of LCs to NF-κB inhibition, we incubated LCs at indicated densities with or without 100nM BAY for 12 hours. Cells were then collected and seeded into methylcellulose for CFU (Supplementary Figure 2). We found that the sensitivity of LCs to NF-κB inhibitor treatment is dependent on cell density. We then treated LCs in high density (HD, 5×10^5^/ml) and low density (LD, 1×10^4^/ml) conditions with indicated dosages of BAY for 12 hours. Cells were then collected for CFU (Figure 2A) or injected into lethally-irradiated receipt mice for transplantation studies (Figure 2B). We found that, consistent with previous studies, LPs (Figure 2A) and LSCs (Figure 2B) are highly sensitive to BAY treatment in LD culture, as demonstrated by CFU assay and *ex vivo* treatment followed by transplantation. BAY treatment induced significant apoptosis in LCs in the LD condition, as shown by cell morphology (Figure 2C) and Annexin-V staining (Figure 2D). *Tnfr^−/−^* LCs are more sensitive to NF-κB inhibitor treatment compared to *WT* LCs in such LD conditions (Figure 2E). However, when cultured in HD conditions, the killing effects of NF-κB inhibition on LPs (Figures 2A, 2E) and LSCs (Figure 1B) were significantly attenuated in both *WT* and *Tnfr^−/−^* LCs. However, the sensitivity of LPs to the NF-κB inhibitor could be largely restored by JNK inhibition (Figure 2E and Supplementary Figure 3), further supporting the notion that, in addition to TNF, other factors secreted by LCs might also provide protection to LPs and LSCs [16, 19] through activation of JNK. To further support this notion, we found that JNK signaling was more highly activated in *WT* LCs than in *Tnfr^−/−^* LCs when cultured in LD condition. However, in HD culture, JNK signaling was comparable between *WT* and *Tnfr^−/−^* LCs (Figure 2F).

### IL1 promoted the growth of LPs independent of TNF

**Figure 3:**
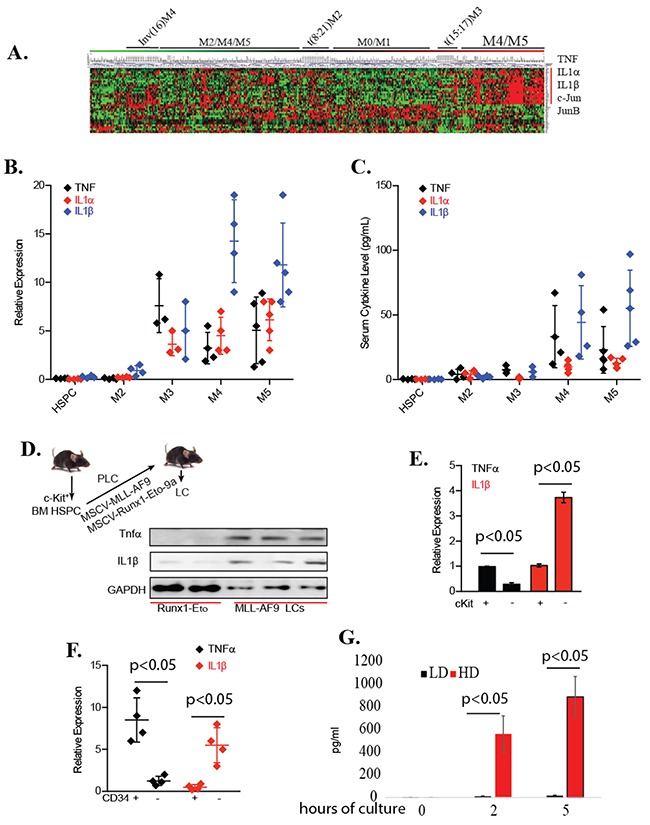
Expression of both TNF and IL1β in many types of LCs **A.** Expression of TNF, TNFR, IL-1, IL-1R, c-JUN and JUNB in primary human AML samples was compared by microarray. **B.** TNF and IL-1 protein levels in PB of AML patients were examined by ELISA. **C.** TNF and IL-1 mRNA in primary AML samples were examined by qRT-PCR assay. **D.** Schematic for development of murine MA9- and Runx1-Eto-9a LCs. TNF and IL1β expression was examined by Western blotting. Each lane was from LCs from a separate transplanted mouse. **E.** Expression of TNF and IL1β in c-kit^+^ LSCs/LPs and c-kitlow^/-^ LBs isolated from murine *MA9-LCs* were examined by qRT-PCR. **F.** Expression of TNF and IL1β in CD34^+^ LSCs/LPs and CD34^low/-^ LBs isolated from 4 primary AML patient samples was examined by qRT-PCR. **G.** *MA9-LCs* were cultured in LD or HD conditions. IL1β concentration was examined in the culture medium at indicated times of incubation.

To search for these other autocrine factors that can activate JNK targets and counteract the anti-AML effects of NF-κB inhibitors, we first analyzed the expression profile of inflammatory cytokines in 580 primary human AML samples in our microarray data (Figure 3A). Although the expression levels of TNF are generally elevated in M3/4/5AML cells compared to normal HSPCs, a subset of M4 and M5 AML samples also express much higher levels of IL1 (both α and β). We confirmed in newly-diagnosed M4/5 subtype AML patients that there were increased levels of pro-inflammatory cytokines TNF and IL-1 in their peripheral blood (Figure 3B), which correlated to the increased TNF and IL1 expression by CD34^+^ tumor cells (Figure 3B). We also determined that *MA9*-transduced murine LCs expressed and produced endogenous TNF and IL1β (Figure 3D). Consistent with a previous study [11], we found that TNF is highly expressed in c-kit^+^ LSCs and LPs (Figure 3E). In addition, we found that IL1β is highly expressed in c-kit^low/-^ partially-differentiated LBs compared to c-kit^+^ LSCs/LPs (Figure 3E). In human primary LCs, TNF was highly expressed in CD34^+^ LSCs and LPs, while IL1β was highly expressed in CD34^-^ LBs (Figure 3F). Significantly higher concentrations of IL1β were detected in LC culture medium when cultured in HD conditions than when cultured in LD conditions (Figure 3G).

**Figure 4:**
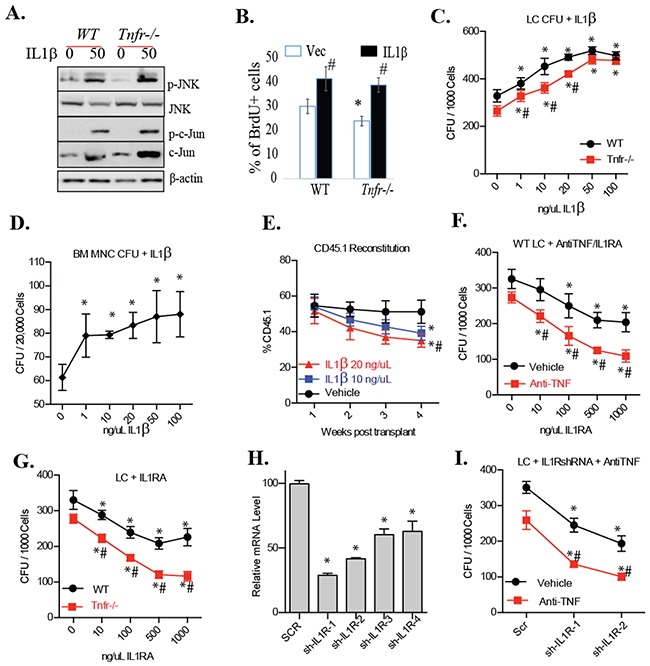
IL-1 promotes the growth of clonogenic LPs **A.** *WT, Tnfr^−/−^, Rip1^−/−^* LCs were treated with IL1β for 15 minutes. JNK activity was examined by Western blotting. **B.** *WT LCs* and *Tnfr*^−/−^ LCs cultured in LD condition (1×104/ml) and treated with 50ng/ml of IL1β. Twelve hours after treatment, cells were treated with 10μM BrdU for an additional 2 hours. Cells were collected for BrdU staining using APC BrdU Flow Kits (BD Pharmingen) following the protocol provided by the vender. * indicates p<0.05 compared to untreated WT LCs. # indicates p<0.05 compared to untreated controls. **C**. *WT* and *Tnfr*^−/−^ LCs were cultured in LD condition (1×104/ml) and treated with increasing concentrations of recombinant IL-1β for 12 hours. Cells were then were seeded for CFU assay. **D.** Normal bone marrow mononuclear cells (MNCs) were treated with increasing concentrations of IL1β for 12 hours and then seeded for CFU assay. **E.** One million bone marrow MNCs were isolated from normal CD45.1+ mice and treated with indicated concentrations of IL-1β for 24 hours. Treated cells were mixed with 1×106 support MNCs (CD45.2+) and transplanted into lethally-irradiated recipient mice (CD45.2+) to evaluate hematopoietic reconstitutive capacity. Five mice were used in each treatment group. **F.** *WT LCs* were cultured in HD condition (5×105/ml) and treated with 20ug/ml of anti-TNF and indicated doses of IL-1RA for 12 hours. Cells were collected and seeded for CFU. **G.** *WT* and *Tnfr*^−/−^ LCs were cultured in HD condition and treated with increasing concentrations of IL-1RA for 12 hours. Cells were collected for CFU assay. H. shRNA knockdown of IL-1 receptor 1 (IL-1R1) in LCs as determined by qRT-PCR. **I.** LCs transduced with shRNAs specific for IL-1R (sh-IL-1R-1 and sh-IL-1R-2) were cultured in HD condition and treated with or without 20ug/ml of anti-TNF. Twelve hours hence, cells were seeded for CFU assay. Scrambled (Scr)-shRNA transductions were studied in parallel as controls. * indicates p<0.05 when compared to 0ng/ml IL1β group (C-E), or 0ng/ml IL1RA groups (**F, G**), or Scr groups (**H, I**). # indicates p<0.05 when compared to *WT* LCs (**C, G**), and vehicle groups (**F, I**) as determined by one-way ANOVA.

We found that IL1β can induce JNK signaling independent of TNF signaling (Figure 4A). To study whether IL1β can promote the growth of LCs independently of TNF, we treated *MA9*-transduced *Tnfr^−/−^* LCs and *MA9*-transduced *Tnfr* wild-type LCs (*WT* LCs hereafter) with IL-1β and measured proliferation and colony formation. We observed that IL1β promotes proliferation (Figure 4B) and colony-forming ability in both *Tnfr^−/−^* and *WT* LCs in a dose-dependent fashion when <50ng/ml of IL1β was used (Figure 4C). IL-1 doses >50 ng/mL did not further enhance CFUs in LCs. Although IL1β also promotes the clone-forming capacity of HSPCs (Figure 4D), we found that it compromises the hematopoietic reconstitutive ability of hematopoietic stem cells (HSCs) as demonstrated by *ex vivo* treatment and transplantation studies (Figure 4E). This suggests that although IL1β promotes the colony-forming ability of normal progenitors, it represses HSC function. We then treated the LCs with a combination of IL1RA, a natural IL1 antagonist, and anti-TNF, a TNF neutralizing antibody. We found that the combined inhibition of IL1β and TNF signaling had an additive, dose-dependent repressive effect on CFU in LCs (Figure 4F). We confirmed this conclusion by treating *Tnfr^−/−^* LCs with IL1RA (Figure 4G). To verify the specificity of IL1RA treatment, we knocked down IL1 receptor-1 (*IL1R*) in LCs by shRNA (Figure 4H). We found that genetic inhibition of *IL1R* also sensitized LCs to anti-TNF treatment (Figure 4I). LCs with *IL1R* knockdown generated far fewer CFUs in the presence of anti-TNF when compared to Scr-shRNA-transduced LCs.

### Combined inhibition of TNF and IL1 sensitized LPs and LSCs to NF-κB inhibition in high-density culture condition

**Figure 5:**
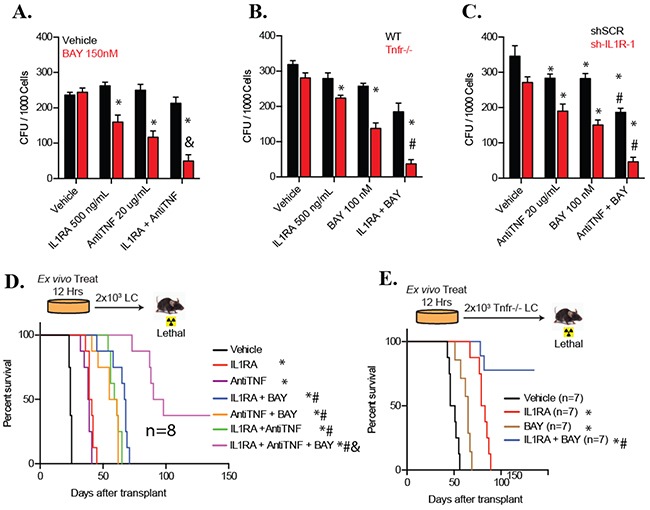
LSCs and LPs with both TNF and IL-1 signaling inactivation are much more sensitive to NF-κB inhibition than are LSCs and LPs with inactivation either of the cytokine signaling alone **A.** WT LCs were cultured in HD condition (5×105/ml) and treated with 20ug/ml of anti-Tnf, 500ng/ml IL-1RA, 150nM BAY individually or in combination for 12 hours, and then seeded into methylcellulose for CFU assay. Vehicle treatment was used as a control. **B.** WT LCs and *Tnfr*^−/−^ LCs in HD condition were treated with 500ng/ml IL-1RA and 100nM of BAY individually or in combination for 12 hours and seeded for CFU assay. **C.** sh-IL-1R-1-transduced LCs (as described in Figure 4G) cultured in HD condition were treated with 100nM BAY or anti-TNF for 12 hours and then seeded for CFU assay. Scrambled shRNA (Scr) transductions were studied in parallel as controls. D. WT LCs were cultured in HD condition (5×105/ml) and treated with 20ug/ml of anti-Tnf, 500ng/ml IL-1RA, 100nM BAY individually or in indicated combinations for 12 hours. Cells were collected and transplanted to assess leukemogenic ability. Vehicle treatment was used as a control. **E.** *Tnfr*^−/−^ LCs were cultured in in HD condition (5×105/ml) and treated with 500ng/ml IL-1RA or 100nM BAY individually or in combination for 12 hours. Cells were collected and transplanted to assess leukemogenic ability. Two thousand initial cells were transplanted into lethally-irradiated recipient mice with BM support cells. Survival of the recipient mice was analyzed by Kaplan-Meier survival graphing. Results shown for (**A-C**) are representative of three independent trials. * indicates p<0.05 when compared to vehicle control; # indicates p<0.05 when compared to individual chemical treatment groups; & indicates p<0.05 when compared to two chemical combination groups. Statistical significance was determined by one-way ANOVA with Bonferonni post-hoc test.

Since both TNF and IL-1 stimulate the activation of JNK [16] (Figure 4A), we treated *WT* LCs in HD condition with IL1RA or anti-TNF individually or in combination for CFU assay in the presence or absence of the NF-κB inhibitor BAY for 12 hours and seeded them for CFU. We found that while IL1RA or anti-TNF alone can repress the CFU of LCs, combination treatment significantly enhances the CFU inhibitory activity of the NF-κB inhibitor, suggesting that co-inactivation of IL1 and TNF signaling can significantly sensitize LCs to NF-κB inhibition in HD culture condition (Figure 5A). We confirmed this conclusion by treating *Tnfr^−/−^* LCs with IL1RA and BAY alone or in combination (Figure 5B). We also further confirmed the conclusion by treating IL1R-knockdown LCs with anti-TNF and BAY alone or in combination (Figure 5C). However, inhibition of both TNF and IL-1R signaling did not enhance the CFU inhibitory activity of the JNK inhibitor, suggesting that TNF and IL-1 are the primary stimuli for JNK in LCs (Supplementary Figure 4).

We then determined that treatment of LCs with IL1RA, anti-TNF, or BAY alone or in the indicated combinations *ex vivo* had effects specific to LSCs by performing *ex vivo* treatment followed by transplantation. After 24 hours of indicated treatments, LCs were collected for cell death analysis and transplanted into recipient mice to observe for leukemia development (Figure 5D). Each mouse was transplanted with 2×10^3^ of the initial LCs. Mice were then observed for leukemia development. A significant increase in cell death was detected in IL1RA, anti-TNF, or BAY single-treated LCs when compared to vehicle treated control; cell death was further increased in any of the two drug combination groups and even further increased in the three drug combination condition (Supplementary Figure 5a). As a consequence, we found that disease onset in mice that had received either IL1RA, anti-TNF, or BAY single-treated LCs was significantly delayed compared to mice receiving vehicle-treated LCs, and was even further delayed in mice receiving LCs treated with any of these two chemical combinations. Most importantly, mice receiving all three chemically-treated LCs survived longest, with 3 out of 8 mice surviving over 135 days without any sign of leukemia (Figure 5D). This study suggested that co-inhibition of TNF and IL-1 signaling significantly enhanced the ability of NF-κB inhibitor to eliminate LSCs. We confirmed these results by combined treatment of *Tnfr^−/−^* LCs with IL1Ra and BAY *ex vivo* (Figure 5E). IL1RA or BAY treatment induced significant cell death in *Tnfr^−/−^* LCs. Significantly more cell death was detected in the IL1RA and BAY combination group (Supplementary Figure 5b). Consistent with this observation, mice receiving BAY or IL1RA-treated *Tnfr^−/−^* LCs required a significantly longer latency for leukemia development than mice receiving vehicle-treated *Tnfr^−/−^* LCs. Mice which had received BAY plus IL1RA treated *Tnfr^−/−^* LCs survived much longer, with 5 out of 8 mice surviving over 135 days without any sign of leukemia. Taking together, our data suggest that inactivation of both IL1 and TNF signaling is more effective in facilitating NF-κB inhibitor-mediated LSC elimination than inactivation of either alone (Figure 5E).

### Combined inhibition of TNF/IL1 and NF-κB repressed leukemia development *in vivo*

**Figure 6:**
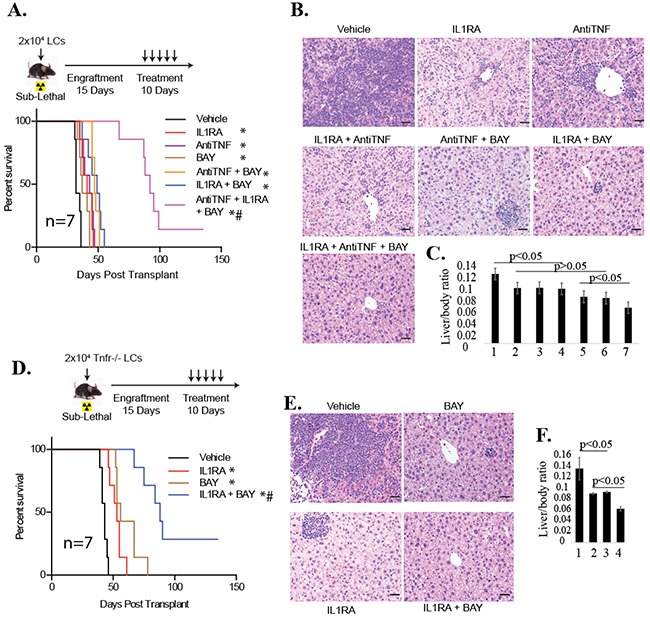
Inhibition of both TNF and IL-1 signaling could more effectively repress leukemia development in vivo than inactivation of either individual signaling pathway when combined with NF-κB inhibitor *WT* LCs **A-C.** or *Tnfr*^−/−^ LCs **D-F.** were transplanted into sub-lethally irradiated recipient mice. Fifteen days following transplantation, mice were treated with 100mg/kg IL-1RA, 10 mg/kg anti-TNF, or 10mg/kg BAY individually or in combinations as indicated every day for 10 days. Survival of the recipient mice was analyzed by Kaplan-Meier survival graphing. Leukemia was confirmed at the time of death for each transplant mouse. *and ** indicate p<0.05 and p<0.01, respectively, when compared to vehicle-treated control, # indicates p<0.05 when compared to single or double treatment controls as determined by log-rank test. Livers were collected in a batch of recipient mice on day 28 for WT LCs **(B, C)** or day 42 for *Tnfr*^−/−^ LCs (**E, F**) of transplantation. LC infiltration was examined by H & E staining of liver sections (**B, E**) and the ratio of liver/body weight was analyzed (**C, F**). In C, 1 stands for Vehicle; 2 for IL1RA; 3 for Anti-TNF; 4 for IL1RA + anti-TNF; 5 for anti-TNF + BAY; 6 for IL1RA + BAY; 7 for IL1RA + anti-TNF+ BAY. In F, 1 stands for Vehicle; 2 for IL1RA; 3 for BAY; 4 for IL1RA + BAY. Three mice from each group were analyzed. *and ** indicate p<0.05 and p<0.01, respectively, when compared to vehicle-treated control.

To evaluate the anti-leukemia effect of the combined treatment *in vivo*, we generated murine leukemia by transplanting *WT* LCs into recipient mice. These mice were treated with the indicated drugs 15 days following transplantation. We found that treatment with any of the single drugs in our study reduced disease burden as shown by reduced liver infiltration (Figure 6B, 6C) and prolonged survival by one week (Figure 6A), with any double inhibitor treatment prolonging survival by another week. Triple inhibition of TNF, IL1, and NF-κB profoundly reduced the disease burden and prolonged survival by a maximum of 90 days (Figure 6A). We confirmed these results by combined treatment of *Tnfr^−/−^* LCs with IL1RA and BAY (Figures 6D-6F). *In vivo* treatment demonstrated that mice which had received *Tnfr^−/−^* LCs survived significantly longer after treatment with BAY and IL1RA in combination than mice treated with either BAY or IL1RA individually (Figure 6D) due to the much more significant repression of disease progression as shown by reduced liver infiltration of LCs (Figure 6C, 6D). Taken together, our data suggest that inactivation of both IL1β and TNF signaling is more effective in facilitating NF-κB inhibitor-induced repression of leukemia development. Our data suggested that the anti-leukemia effect of NF-κB inhibition *in vivo* can be significantly enhanced by inhibition of either TNF or IL1 signaling alone, and can be further promoted by co-inhibition of both TNF and IL1 signaling (Figure 6).

### Inactivation of individual AP1 factors reduced colony-forming ability and promoted the sensitivity of LCs to NF-κB inhibition

**Figure 7:**
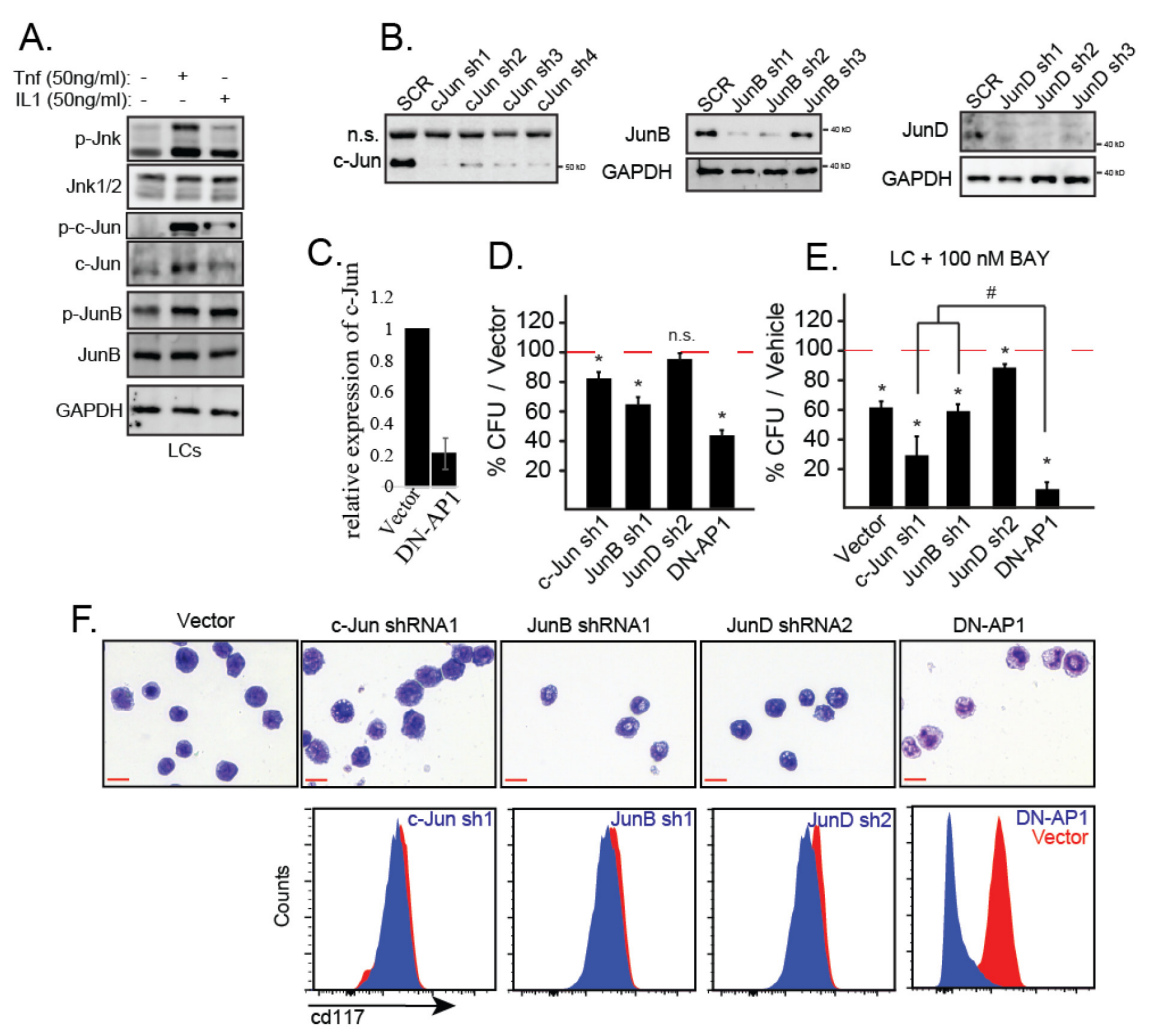
IL-1 stimulates the activation of JNK-AP1 signaling in LCs **A.** WT LCs were treated with TNF or IL1β for 15 minutes. The activity of JNK signaling was examined by detecting the levels of p-JNK, p-Jun, p-JunB and p-JunD. **B.** LCs were transduced with shRNAs specific for c-Jun, JunB or JunD; gene knockdown efficiency was determined by Western blotting. **C.** The effect of DN-AP1 transduction was verified by qRT-PCR to detect downregulation of c-*Jun*. **D.** Clonogenic capacity of gene knockdown LCs was compared to Scr shRNA-transduced LCs and DN-AP1-transduced LCs by CFU assay. **E.** The sensitivity of gene knockdown LCs to NF-κB inhibitor treatment was compared to Scr shRNA-transduced LCs and DN-AP1-transduced LCs by CFU assay. **F.** The morphology and CD117 surface expression were compared among the gene knockdown LCs, Scr shRNA-transduced LCs and DN-AP1-transduced LCs after 72 hours of suspension culture. **A, B, C, F** are representative of three independent trials. * indicates p<0.05 when compared with Scr-ShRNA or vector-transduced LCs as determined by one-way ANOVA with Bonferroni's post-hoc test. # indicates p<0.05 between compared groups as determined by one-way ANOVA with Bonferroni's post-hoc test.

We have reported that TNF induces necroptosis/apoptosis in normal HSPCs by stimulating a prolonged activation of JNK signaling. However, in LCs, the expression of MKP5 turns off JNK signaling shortly after its activation. Thus, TNF induces only a transient JNK activation in LCs [16]. Such transitory activation of JNK promotes the proliferation and survival of LCs by activating downstream transcription factors, such as the AP1 family. Upon stimulation with TNF or IL1β, c-Jun, JunB and JunD were activated in LCs as shown by increased phosphorylation of these proteins (Figure 7A). To determine which AP1 component responds to TNF/IL1-induced survival and proliferation of LCs, we transduced LCs with shRNA specific for *c-Jun*, *JunB* and *JunD* respectively. The knockdown efficiencies of target genes were examined by Western blotting (Figure 7B). We then selected the LCs with the most efficient target gene knockdown for functional studies. Dominant negative (DN)-AP1 is a mutant form of the *c-Jun* gene repressing most of the activity of AP1. The effect of DN-AP1 was verified by down-regulation of *c-Jun*, a well-documented AP1 target gene (Figure 7C). Using CFU assay, we demonstrated that inactivation of *c-Jun* and *JunB* but not *JunD* repressed the clonogenic capacity of LCs (Figure 7D). LCs with either *c-Jun* or *JunB* knockdown showed increased sensitivity to NB-κB inhibitor treatment compared to Scr-shRNA-transduced control (Figure 7E). *DN-AP1* transduction showed much stronger effects in both repressing clonogenic capacity and enhancing NF-κB inhibitor sensitivity than knockdown any of the individual AP1 family members (Figure 7D–7E). Most interestingly, LSCs and LPs in *MA9*-leukemia express CD117 and lose this surface marker during differentiation. We found that *DN-AP1*-transduced LCs showed a differentiated phenotype as demonstrated by both cell morphology and reduced CD117 expression (Figure 7F). Therefore, we predicted that multiple AP1 family members might be involved in regulating proliferation, survival and differentiation of LCs downstream of JNK.

### Combined inhibition of TNF and IL1 potentiated NF-κB inhibitor in repressing colony growth of primary human LCs

**Figure 8:**
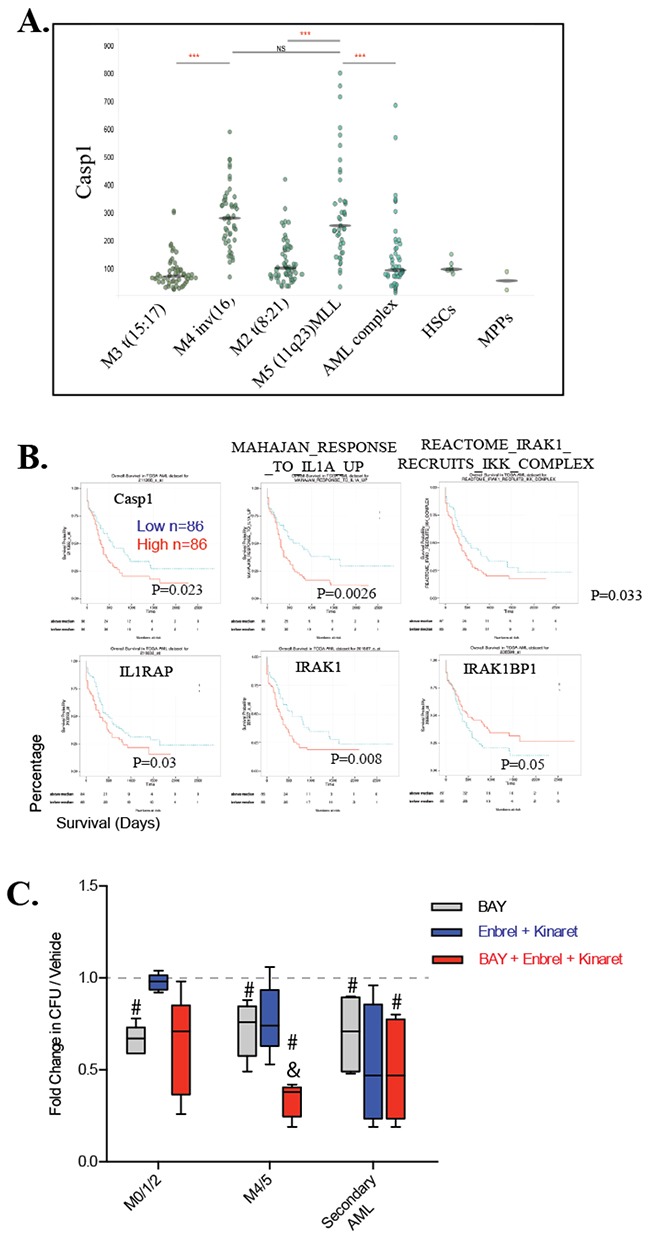
M4/5 AML is highly susceptible to combined TNF/IL-1 and NF-κB inhibition treatment **A.** High levels of expression of Caspase 1 in M4 and M5 subtypes of AML samples as shown by analysis of the TCGA database. **B.** Correlation of IL1-IRAK signaling to patient outcome was examined by analyzing the TCGA database. **C.** Primary AML cells isolated from patient peripheral blood or bone marrow samples were treated with indicated drugs at indicated doses in methylcellulose for CFU assay. Colonies were read after 14 days. Results were normalized to vehicle-treated control for each patient sample. Each dot represents one patient, n=5 for each group. Horizontal bars represent mean, vertical bars represent one SD. *** in A indicates p<0.05 when compared to indicated groups; # indicates p<0.05 when compared to vehicle group; & indicates p<0.05 when compared to other groups.

IL1β induces TNF-independent activation of JNK signaling through the RIP1-MYD88-IRAK pathway [20, 21]. Caspase-1 (CASP1) is the IL1 converting enzyme (ICE) which is required for IL1β secretion and activity. CASP1 is highly expressed in the M4 and M5 subtypes of AML cells (Figure 8A), levels of which are correlated to poorer patient outcome (Figure 8B) as shown by analysis of the AGCT database. Increased expression of IL1RAP and IRAK1 as well as “elevated IRAK1-recruit IKK complex and Mahajan_response_to_IL1A pathway” is also associated with poorer prognosis of AML patients, while the increased expression of IRAK1BP1, a negative regulator of IRAK1 signaling, is correlated to better prognosis (Figure 8B).

To determine whether inhibition of both TNF and IL1 can also promote the anti-leukemic ability of NF-κB inhibition in primary human AML, we treated primary human LCs freshly isolated from peripheral blood of AML patients with BAY and TNF/IL-1 antagonists (*Enbrel*® and *Kinaret*®, respectively) individually or in combination in methylcellulose for CFU assay. We found that combined inhibition of NF-κB and TNF/IL1 did not have an additive repressive effects on CFU in M0/1/2 subtypes of LCs, but synergized in M4/5 subtypes of LCs as well as LCs from AML patients secondary to MDS or therapy for other cancers (Figure 8C).

## DISCUSSION

The inflammatory reaction has been described as a critical component of the tumor environment in many solid tumors. In these tumor tissues, chemokines secreted by malignant cells induce the infiltration of many types of hematopoietic/immune cells (such as macrophages, myeloid-derived suppressor cells, NK cells and T/B-lymphocytes) to the tumor tissues. Most of the tumor-infiltrating hematopoietic/immune cells stimulate a persistent cycle of damage and repair in tumor tissue to generate a tumor-promoting inflammation by producing tumor-supporting cytokines including TNF, IL1, and IL6. Thus, such cells and the inflammatory cytokines and signals generated by them have been proposed to be critical targets for anti-tumor therapy [22–31]. However, almost all of the previous studies evaluated anti-tumor activity by targeting these cytokines and their signaling pathways individually. In fact, signals stimulated by these cytokines are not functionally independent. Most of these cytokines share some common signaling pathways and are functionally compensatory. Thus inactivation of one cytokine-stimulated signal in most cases is insufficient to repress tumor growth due to the compensation of signaling stimulated by other cytokines. Our studies suggest that removing at least TNF and IL1 is necessary to maximally eliminate LCs, and especially for removing LSCs.

Consistent with earlier studies [32, 33], we found that the expression of TNF and IL1 is elevated in many subtypes of AMLs. The levels of TNF and IL1 are increased and correlated to poor prognosis of AML patients, especially older adults [5, 32, 33]. TNF is primarily produced by LSCs/LPs and it promotes the survival and proliferation of LSCs/LPs in an autocrine fashion [11, 16], while IL1β is mainly produced by LBs (Figure 3E, 3F), and also enhances the survival and proliferation of LSCs/LPs in a dosage-dependent paracrine manner. This might explain why cell density is so important to detect the compensatory effect of IL1 on TNF signaling inactivation.

An important role for IL1 in the pathogenesis of AML has be proposed in early studies. It was demonstrated that IL1 promotes the colony-forming ability of LCs by stimulating the activation of downstream signaling and the secretion of hematopoietic cytokines such as GM-CSF in LCs and stromal cells [34]. IL1RAP, a well-known co-receptor of IL1R, is highly expressed in many AML patient samples and may be involved in LSC self-renewal [35, 36]. In addition, elevated phospho-IRAK1, a hallmark of activated IL1 signaling, is detected in advanced MDS and AML patient samples, and has been suggested as an anti-AML target [37]. The expression levels of many other key components of the IL1 signaling pathway are also increased in AML patient samples, and such increases indicate a poorer patient prognosis (Figure 8). Thus the IL1 pathway has been described as a critical therapeutic target for AML. However, despite of the effective repressive role of IL1 antagonists (such as sIL-R or IL1RA) on the growth of LCs in *in vitro* culture [38–40], inhibition of IL1 signaling might not be able to successfully repress AML *in vivo* due to compensation by TNF-stimulated signaling.

Similar to TNF, IL1 stimulates the activation of both JNK and NF-κB signaling [41]. Balanced JNK and NF-κB signaling is critical for the proper response of normal tissue cells to inflammatory cytokine-stimulated reactions [42]. JNK induces pro-apoptotic activity in most normal tissues, which causes tissue damage and even tumor development when NF-κB signaling is inhibited. However, in LCs, JNK mediates a proliferation/survival signaling parallel to NF-κB signaling [16, 43]. Our study suggested a mutual compensation of IL1 and TNF activity in LCs by stimulating both JNK and NF-κB signaling.

Our studies suggested that combined inhibition of JNK and NF-κB signaling might be a better treatment for TNF and IL1-expressing M4/5 subtypes of AML [16]. Interestingly, we found that such combination treatment was also effective in LCs isolated from patients with therapy-related AMLs (Figure 8C). Such AMLs are less responsive to any of the current therapies and portend a poor prognosis. MLL rearrangements are commonly detected in such AML samples [44]. It will be important to determine whether JNK and NF-κB inhibitor combination treatment can effectively repress therapy-related AML *in vivo*. In addition, although our *in vitro* study suggested that the TNF/IL1 low-expressing LCs are less responsive to JNK and NF-κB inhibitor treatment, we predict that our combined inhibitor treatment might also benefit these AML patients when combined with standard therapies because TNF and IL1 can be produced by bone marrow niche cells during chemotherapy or radiation therapy. In fact, accumulated data suggest that in AML patients, the HSC-supporting niches are converted into inflammatory leukemia-promoting niches [45]. Such an inflammatory niche environment plays critical roles in leukemia drug-resistance by protecting LSCs from chemotherapy [46, 47]. Whether or not TNF/IL1-induced JNK/NF-κB signaling contributes to the development of drug resistance needs to be further verified.

Multiple potential NF-κB inhibitors such as the proteasome inhibitor bortezomib and the natural compound parthenolide have been successfully used in the clinical treatment setting for multiple myeloma and are being evaluated in clinical trials for AML treatment in combination with standard chemotherapeutic drugs [48, 49]. Unfortunately, clinical grade JNK inhibitors are not available. We found that in these TNF/IL1-expressing AML cells, as is the case with inactivation of JNK signaling, inhibition of both IL1 and TNF represses growth and significantly sensitizes clonogenic LPs and leukemogenic LSCs to NF-κB inhibitor treatment. Given that both TNF and IL1 antagonists are commonly used clinically to treat rheumatoid arthritis and autoimmune diseases, and have been confirmed to be safe, we speculate that we might able to use these FDA-approved TNF/IL1 antagonists and NF-κB inhibitors to evaluate our novel treatment approach in AML patients. In addition, oral and intestinal mucositis are severe pathologic conditions affecting most patients treated with standard chemotherapy or radiation therapy [50–54]. TNF/IL1-mediated inflammation has been determined to be responsible for such side effects.

## MATERIALS AND METHODS

### Mice

The C57/Bl6J mice used as transplantation recipients in this study were purchased from the Jackson Laboratory and maintained in the Department of Comparative Medicine, Loyola University Chicago. All experiments using mice were performed according to the guidelines of Loyola University Medical Center and were approved by the Loyola University Institutional Animal Care and Use Committee.

### Reagents

Recombinant murine-IL-3 (rm-IL-3), rm-IL-6, rm-SCF and rm-GM-CSF were purchased from eBioscience (San Diego). Recombinant human IL-3, IL-6, SCF, Flt-3, and TPO were obtained from Humanzyme. TNFα was purchased from BD Biosciences. BAY11-7085 and SP600125 small molecule inhibitors were purchased from Millipore. Cell lysis buffer (10×) was obtained from Cell Signaling, and supplemented with proteinase inhibitors and phosphatase inhibitors (Roche Diagnostics). GAPDH antibodies were obtained from Santa Cruz Biotechnology. c-Jun, JunB, and JunD primary and requisite secondary antibodies were also obtained from Cell Signaling. Tri-reagent used for RNA extraction was purchased from Sigma Aldrich. *Etanercept*® and *Kinaret*® were graciously provided by the hematology/oncology clinic at Loyola University Chicago. Methylcellulose for CFU assays was purchased from StemCell Technologies. Anti-CD11b and Anti-CD117 antibodies were purchased from eBioscience. Wright-Geimsa solutions were purchased from Exaxol (Clearwater, FL).

### Generation of murine leukemia cell lines

*MLL-AF9* murine LCs were generated as previously described [16]. Briefly, CD117^+^ HSPCs were isolated from *WT* and *Tnfr^−/−^* mice (knockout of both *Tnfr 1* and *2*) and infected with *MLL-AF9*-neo-*expressing retrovirus*. Infected cells were transplanted into lethally-irradiated recipient mice to generate leukemic mice. *WT* and *Tnfr^−/−^* LCs isolated from spleens and BM of the corresponding leukemic mice were used in our studies.

### Colony-forming unit assay

LCs and BM cells were seeded into *MethoCult GF M3434*^®^ (StemCell) at 1000 cells/mL (LCs) or 20,000 cells/mL (BM HSPC), incubated at 37°C, 100% humidity, and 5% CO_2_ for 7 days (LC) or 10 days (HSPC cells). Numbers of colonies were counted according to the manufacturer's instructions. Triplicate experiments were performed in all of our studies. All data were verified by three individual experiments. Primary AML patient samples were seeded into *MethoCult 4035 Optimum* without EPO and incubated at 37°C, 100% humidity, and 5% CO_2_. Colonies were read 14 days following seeding.

### Retroviral infection

High-titer retrovirus was produced by co-transfecting Phoenix cells with a retroviral vector containing the indicated genes together with packaging vectors using *Calphos Mammalian Transfection Kit* (Clontech). Retroviral supernatants were harvested 24 and 48 hours after transfection. MSCV-MLL-AF9-neo was kindly provided by Nancy Zeleznik-Le of Loyola University Chicago, and pMieg-DN-AP1 was obtained from Addgene. Viruses were generated using these retroviral vectors. LCs were transduced with such virus-expressing genes of interest by spinoculation at 32°C, 2000 rpm for 4 hours. Transduced cells were purified by FACS for Western blotting and CFU assay.

### *Ex vivo* transplantation

Ten thousand LCs (CD45.2^+^) were plated in each well in a suspension culture and treated with indicated doses of BAY11-7085, SP600125, anti-TNF antibody (Amgen), and IL-1Ra (Anakinra, Amgen) in indicated combinations for 12 hours. All cells in each well were harvested and mixed with 10^6^ support BM cells (CD45.1^+^). The mixed cells were equally divided and transplanted into 10 lethally-irradiated (350cGy) recipient mice (CD45.1^+^). Mice were monitored for leukemia development by observing for symptoms: hunched body, significant weight loss, or hind-limb paralysis. Leukemia was confirmed by examining CD45.2^+^ LCs in PB, spleen and BM, as well as liver and kidney infiltration.

### *In vivo* transplantation and treatment

Two thousand LCs were transplanted into sub-lethally irradiated (350cGy) C57BL6/J mice via tail vein injection. Twenty days after transplantation, mice were treated with 10mg/kg *InVivo*MAb anti m-TNFα (*BioXcell*), 10 mg/kg BAY11-7085, 30 mg/kg SP600125, or IL-1RA (Anakinra, Amgen) individually or in combinations every day for 10 days. Mice were monitored for leukemia development by observing for lethargy, paralysis, significant weight loss and/or enlarged abdomen. Leukemia was verified after the mice were sacrificed by examining for infiltration of LCs into livers, lungs and spleens.

### shRNA knockdown

LCs were transduced with retrovirus-expressing shRNAs (Origene) specifically targeted to *c-Jun* (TG501139), *JunB* (TG516091), *JunD* (TG501140), and *IL-1R* (TG501076). The transduced cells were selected for one week using puromycin (c-Jun shRNA) or by GFP sorting (JunB, JunD, IL-1R) to obtain stably transduced cells. Knockdown efficiency was examined by Western blotting (c-Jun, JunB, JunD), or RT-PCR (IL-1R). Scrambled *shRNAs* were transduced and studied in parallel as controls.

### Primary human AML samples

Peripheral blood samples from AML patients were obtained from the clinic at Loyola University Medical Center in accordance with the IRB protocol. Leukemic blasts in PB of all patients were 30-90% when samples were collected. Samples were processed for mono-nuclear cells (MNC) by Ficoll-paque gradient centrifugation. A portion of MNC was used for RNA extraction and TNF expression analysis; another fraction of MNC was plated in *StemSpan* serum-free medium (StemCell) supplemented with recombinant human SCF (100 ng/mL), Flt-3L (100 ng/mL), TPO (20 ng/mL), IL-6 (20 ng/mL), and IL-3 (20 ng/mL). Cytokines were obtained from Humanzyme. Following overnight culturing, 3×10^5^ cells from each sample were harvested and treated with the indicated doses of BAY11-7085, anti-TNF (*Etanercept*®), or anti-IL1 (*Kinaret*®), and plated into methylcellulose (StemCell) for CFU assay. Colonies were read after 14 days. In addition, serum was collected from the same patients for examination of TNF and other cytokines.

### Liver/body ratio and liver histologic analysis

Livers were collected from mice at indicated time points and weighed. The ratio of liver/body weight was calculated. Livers were fixed in zinc formalin at room temperature for 3 days. Tissues were then transferred into 70% ethanol until embedding. Embedding and cutting of sections were performed according to standard protocols of the Pathology Department, Loyola University Medical Center. Slides were then stained with H&E.

### Annexin-V and 7-AAD staining to analyze for apoptosis

LCs with indicated treatments were collected and stained with allophycocyanin-conjugated Annexin-V followed by 7-amino-actinomycin D (7-AAD) staining in binding buffer following the manufacturer's instructions (BD Biosciences). Death of infected cells was examined for the percentages of Annexin-V^+^ and Annexin-V^+^/7AAD^+^ cells by flow cytometry.

### Statistical analysis

One-way ANOVA with Bonferonni post-hoc test was used for all *in vitro* comparisons, except where otherwise noted. Log-rank test was used for all *in vivo* comparisons. All statistical analyses were performed using *Graphpad Prism* software.

## SUPPLEMENTARY MATERIALS FIGURES AND TABLES


