# Supplementary material for: Sensitizing leukemia stem cells to NF-κB inhibitor treatment in vivo by inactivation of both TNF and IL-1 signaling
Source: Oncotarget. 2016 Dec 26;8(5):8420–35. doi: 10.18632/oncotarget.14220 (PMC5352411; doi:10.18632/oncotarget.14220)
Supplement: Supplementary file 1 [file oncotarget-08-8420-s001.pdf]

# Sensitizing leukemia stem cells to NF-κB inhibitor treatment *in vivo* by inactivation of both TNF and IL-1 signaling

## SUPPLEMENTARY FIGURES

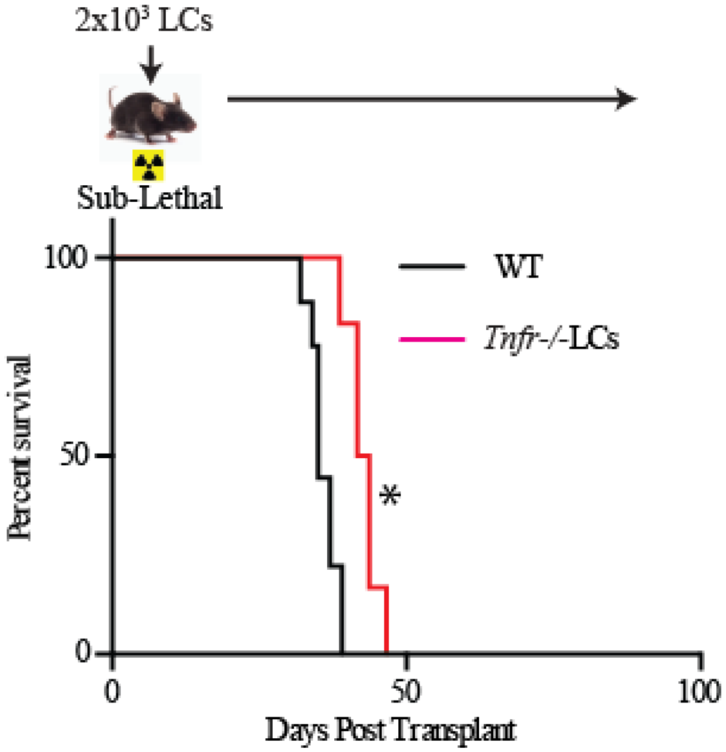


**Supplementary Figure 1. Delayed leukemia development in mice which had received *Tnfr*$^{-/-}$ LCs compared to mice receiving *WT* LCs.** *WT* and *Tnfr*$^{-/-}$ LCs were transplanted into recipient mice to observe for leukemia development. Survival of recipient mice was analyzed by Kaplan-Meier survival graphing. * indicates $p<0.05$ when compared to *WT* controls.

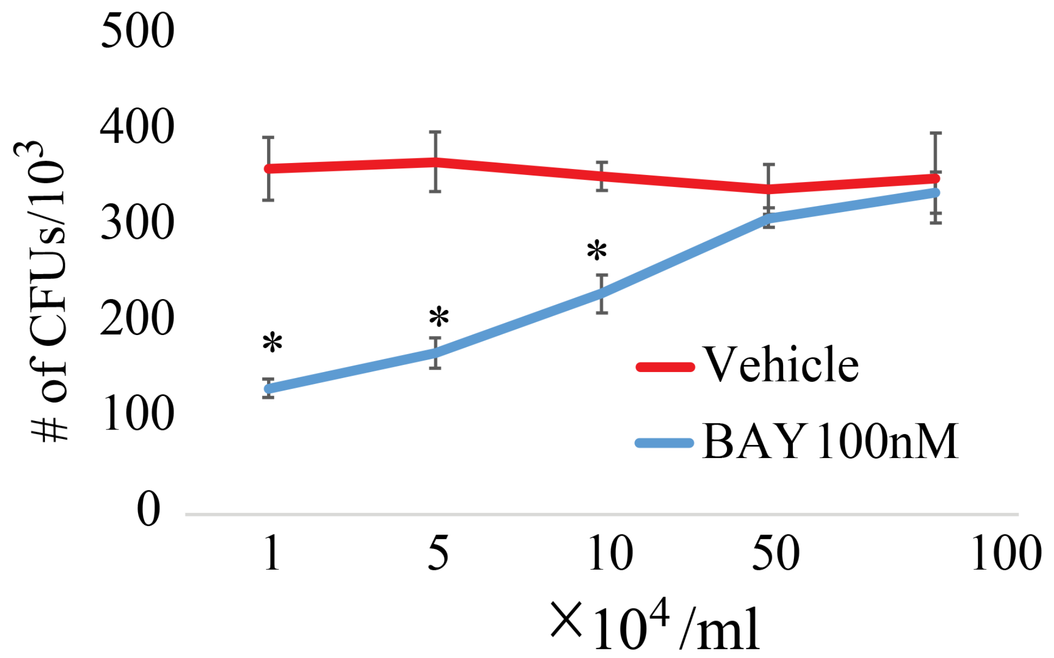


**Supplementary Figure 2. Density-related response of LPs to NF-κB inhibition.** *WT* LCs were cultured at the indicated density for 12 hours with 100nM BAY and then collected for colony-forming assay. The number of cells seeded per plate was based on the number of initial treated cells. Results shown are indicative of three independent trials. * indicates $p<0.05$ when compared to vehicle controls.

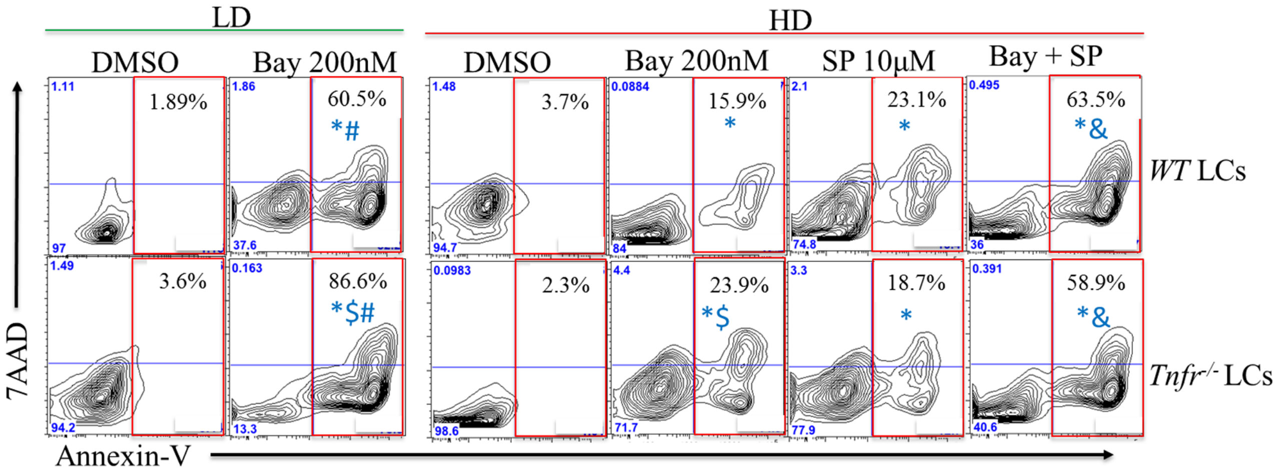


**Supplementary Figure 3. Inhibition of JNK resensitizes LCs to BAY treatment in HD culture condition.** (Correlated to Figure 2e). *WT* and *Tnfr*$^{-/-}$ LCs were incubated at LD ($1\times10^4$/ml) or HD ($5\times10^5$/ml) and treated with 200nM BAY, 10μM SP6 or both in combination for 12 hours and then collected for cell death analysis by Annexin-V/7AAD staining. Vehicle treatment was used as a control. * indicates $p<0.05$ when compared to vehicle controls. $ indicates $p<0.05$ when compared to *WT* LCs. # indicates $p<0.05$ when compared to HD groups with the same treatment. & indicates $p<0.05$ when compared to HD groups with BAY or SP individual treatment.

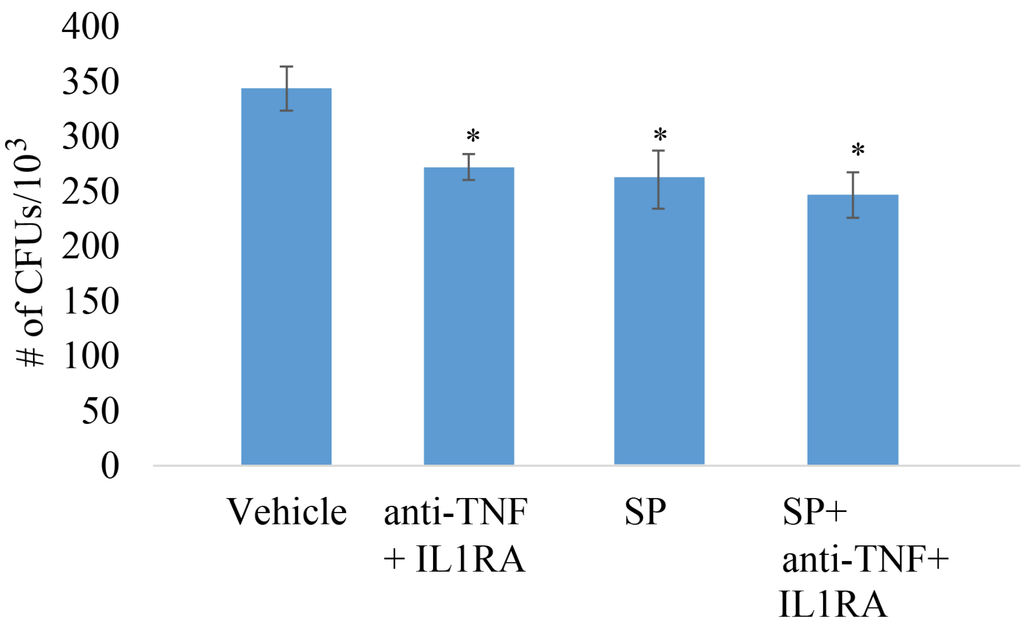


**Supplementary Figure 4. Co-inhibition of TNF and IL1 signaling did not further enhance the repressive ability of JNK inhibitor on LPs.** *WT* LCs were cultured in HD and treated with 10μM SP6, anti-TNF + IL1RA or anti-TNF + IL1RA + 10μM SP6. Cells were collected after 12 hours of treatment for colony-forming assay. The number cells seeded per plate was based on the number of initial treated cells. Results shown are indicative of three independent trials. * indicates $p<0.05$ when compared to vehicle controls.

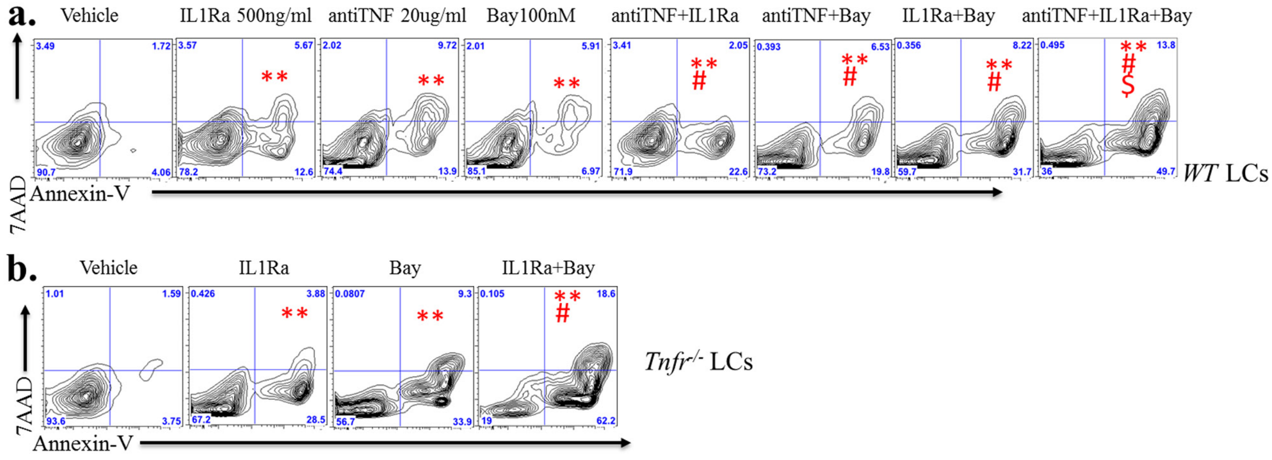


**Supplementary Figure 5. Inhibition of both TNF and IL1 signaling promoted NF-κB inhibitor-induced cell death in LCs (correlated to data in Figure 5d, e).** *WT* LCs **a.** and *Tnfr*$^{-/-}$ LCs **b**. were cultured in HD ($5\times10^5$/ml) condition and treated with indicated treatment for 12 hours. Cell death was examined by Annexin-V and 7AAD staining followed by flow cytometric analysis. ** indicates $p<0.01$ when compared to vehicle controls. # indicates $p<0.05$ when compared to individual drug treatment. $ indicates $p<0.05$ when compared to two drug combination groups.
